# Temporal dynamics in the association between depression and dementia: an umbrella review and meta-analysis

**DOI:** 10.1016/j.eclinm.2025.103266

**Published:** 2025-05-29

**Authors:** Jacob Brain, Maha Alshahrani, Aysegul Humeyra Kafadar, Eugene YH. Tang, Elissa Burton, Leanne Greene, Deborah Turnbull, Bronwyn Myers, Aliya Naheed, Mario Siervo, Phillip J. Tully, Blossom CM. Stephan

**Affiliations:** a Institute of Mental Health, The University of Nottingham Medical School, Nottingham, NG7 2UH, UK; b Freemasons Foundation Centre for Men's Health, Discipline of Medicine, School of Psychology, The University of Adelaide, Adelaide, SA, Australia; c Dementia Centre of Excellence, Curtin enAble Institute, Faculty of Health Sciences, Curtin University, Perth, Australia; d Saudi Arabian Cultural Mission in Australia, Australia; e School of Population Health, Curtin University, Perth, WA 6102, Australia; f Research Centre of Advanced Materials, King Khalid University, Abha, Saudi Arabia; g Population Health Sciences Institute, Faculty of Medical Sciences, Newcastle University, Newcastle upon Tyne, United Kingdom; h Curtin School of Allied Health, Faculty of Health Sciences, Curtin University, Perth, WA, 6102, Australia; i Department of Health and Community Sciences, University of Exeter Medical School, Faculty of Health and Life Sciences, University of Exeter, Exeter, EX1 2LU, UK; j Curtin EnAble Institute, Faculty of Health Sciences, Curtin University, Perth, WA, 6102, Australia; k Mental Health, Alcohol, Substance Use and Tobacco Research Unit, South African Medical Research Council, Cape Town, South Africa; l Non-Communicable Diseases, Nutrition Research Division, International Centre for Diarrhoeal Disease Research, Bangladesh; m School of Psychology, Deakin University, Burwood, VIC, Australia

**Keywords:** Dementia, Depression, Risk factors, Review, Meta-analysis

## Abstract

**Background:**

Identifying modifiable risk factors is crucial for dementia prevention, a global health concern. Depression is considered a risk factor for dementia, but the temporal dynamics across the life course remain inconclusive. Therefore, we aimed to systematically assess the relationship between the timing of depression assessment and risk of all-cause late-life dementia.

**Methods:**

We conducted an umbrella review and meta-analysis to assess incident dementia in individuals with non-current history of depression. PubMed and Ovid Embase, MEDLINE, and PsycInfo were searched from inception up to February 17, 2025. Systematic reviews with meta-analyses investigating the association between depression and incident late-life dementia were included. From eligible reviews, we also extracted data from studies reporting dementia risk as hazard ratios (HRs), analysing the timing of depression measurement using random-effects models for meta-analysis. This study is registered with PROSPERO, CRD42021249706.

**Findings:**

Of the 7763 records identified, nine reviews were eligible for inclusion of the umbrella review. One review was judged to be of moderate quality, while the others were either low (*n* = 3) or critically low (*n* = 5). For our meta-analyses, 18 studies reporting depression onset in later life (*n* = 901,762 participants, *n* = 7595 incident dementia cases) and seven studies on depression assessed during midlife (*n* ≥ 2,501,269 participants, *n* ≥ 276,929 incident dementia cases) were included. All studies in the meta-analyses were deemed to be of good quality, with no strong evidence of publication bias. Pooled HRs indicated depression present in late-life (HR 1.95, 95% CI: 1.68–2.26; *I*^*2*^ = 77.5%) and midlife (HR 1.56, 95% CI: 1.12–2.18; *I*^*2*^ = 97.5%) significantly increased risk of all-cause dementia.

**Interpretation:**

The findings suggest that depression across the life course may increase dementia risk; however, substantial heterogeneity and review quality should be considered when interpreting the strength of this evidence. A life course approach to the treatment and prevention of depression may help reduce the burden of dementia, but this will require scaling up access to effective mental health care for vulnerable populations. Further research is needed to clarify if the stronger late-life association reflects depression as an immediate risk factor or an early manifestation of neurodegenerative processes.

**Funding:**

10.13039/501100000272National Institute for Health and Care Research, 10.13039/100014013UK Research and Innovation, and Saudi Arabian Cultural Mission.

**Research in context**

**Evidence before this study**

Between January 1, 2020, and December 31, 2024, we searched PubMed, Embase, and PsycInfo via Ovid for umbrella reviews, systematic reviews, and meta-analyses investigating the association between depression and the risk of dementia. We used the terms “depression,” “depressive symptoms,” “dementia,” “Alzheimer,” “vascular dementia,” “umbrella review,” “overview of reviews”, “systematic review,” and “meta-analysis” in the title and abstract fields, with no language restrictions. Although numerous meta-analyses have reported significant associations between depression and subsequent dementia, we identified only one relevant umbrella review that evaluated multiple mental disorders, including depression, in relation to dementia risk. While that review reported high-strength evidence linking late-life depression (defined as age ≥50 years) to increased dementia risk, it did not stratify results by life stage nor explore methodological sources of heterogeneity such as follow-up duration, population characteristics, or exposure definitions. To date, no umbrella review has systematically assessed how the timing of depression influences the risk of developing dementia.

**Added value of this study**

This umbrella review and meta-analysis is based on data extracted from the nine eligible reviews, including 18 individual studies focused on late-life depression (>901,762 participants and >7595 incident dementia cases), and seven individual studies focused on midlife depression (>2,501,296 participants and >276,929 incident dementia cases) with late-life all-cause dementia as the outcome. It is the first study to provide pooled evidence focused specifically on the complex temporal relationship between depression assessment and late-life dementia. The results show that the timing of depression assessment, i.e., midlife and late-life, are both significantly associated with an increased risk of all-cause dementia, with varying strength of association. Moreover, the observed substantial heterogeneity might reflect interactions with other factors such as vascular changes, neuroinflammation, or disease co-morbidity.

**Implications of all the available evidence**

These findings contribute to the evidence supporting depression as a potentially modifiable risk factor for dementia, while highlighting a complex temporal gradient in this association. Given the substantial public health implications, there is an urgent need to scale up depression detection and treatment efforts, particularly in primary care settings and for older populations. Expanding access to mental health services through integrating mental health screening and referral to treatment into routine health assessments for mid-life and older adults could be pivotal steps in mitigating dementia risk.

## Introduction

Dementia is a significant public health priority, affecting over 57 million people globally.^1^ With no cure, the identification and treatment of modifiable factors to reduce dementia risk, such as depression, is an important public health priority.^2^, ^3^, ^4^ Depression, particularly late-life onset depression, has been linked to late-life (i.e., aged ≥65 years) dementia.^5^^,^^6^ However, recent evidence suggests that the risk is conferred earlier than previously thought. The 2020 Lancet Commission on Dementia identified late-life depression as a significant modifiable risk factor for dementia, with a global population attributable fraction (PAF) of 3.9%.^7^ In contrast, the 2024 Commission reclassified depression as a midlife risk factor for dementia with a PAF of 3.0%.^3^ The Commission acknowledged the potential bi-directional relationship between depression and dementia, with depression potentially a prodromal manifestation of underlying neurodegeneration and an independent risk factor for incident dementia.^7^ However, whether depression primarily acts as a risk factor, a prodrome, or shares common mechanisms with dementia remain unclear.

The potential mechanisms linking depression to dementia are complex and may include chronic inflammation, hypothalamic–pituitary–adrenal axis dysregulation, vascular changes, alterations to neurotrophic factors and neurotransmitter imbalances.^8^^,^^9^ Shared genetic and lifestyle related changes may also drive risk.^8^^,^^10^ Moreover, midlife and late-life depression may have distinct roles in dementia risk, with shorter intervals between depression and dementia possibly reflecting prodromal changes.^11^

Prior meta-analyses^3^^,^^7^ and epidemiological studies^12^ reveal heterogeneity in the association between depression and incident dementia risk. This inconsistency may be attributed to methodological differences in depression and dementia diagnostic assessments, study populations, follow-up duration, and consideration of effect modifiers such as age, sex, and medication use. Therefore, this umbrella review and meta-analysis aims to synthesise existing evidence on the relationship between depression and dementia, with an emphasis on the temporal dynamics of depression assessment and dementia risk.

## Methods

### Search strategy and selection criteria

This umbrella review was conducted according to PRISMA guidelines^13^ and registered on PROSPERO (ID: CRD42021249706).

PubMed and Embase, MEDLINE, and PsycInfo (via Ovid) were searched from database inception to the 17th of February 2025 using a combination of terms and filters for depression, dementia (including sub-types such as Alzheimer's disease [AD] and vascular dementia [VaD]) and review (systematic and meta-analysis) (Appendix pp2).

Eligible articles were systematic reviews with meta-analyses that examined the association between depression (regardless of when depression was assessed and treatment status) and the incident risk of late-life dementia (i.e., ≥65 years) including all-cause dementia and its subtypes. There were no restrictions on language, study location or setting. Book chapters, commentaries, non-systematic reviews, trials, and conference abstracts were excluded. Reviews that included prevalent and incident studies were included, however, only the incident results were extracted. Reviews that included risk of both mild cognitive impairment and dementia were included if the results for the dementia risk outcome were reported separately.

Initial literature selection was based on double-screening titles and abstracts, undertaken independently by five authors (JB, MA, MS, BCMS, EYHT). Following this, full-texts that met the inclusion criteria were assessed independently by three authors (JB, MA, BCMS). Backward citation chasing was undertaken to identify any papers missed in the electronic search. Any disputes were resolved by discussion until consensus was reached.

Two authors (JB, AHK) independently used the AMSTAR 2 (A Measurement Tool to Assess Systematic Reviews 2) to critically appraise the quality of included reviews.^14^ To assess the quality of studies included in the meta-analysis, two authors (JB, LG) independently used the Newcastle–Ottawa Scales (NOS) for cohort and case–control studies,^15^ with disagreements resolved by discussion.

Three authors (JB, MA, LG) independently completed data extraction which was checked by a fourth author (AHK). Extraction included first author, publication year, methods (databases searched, time limits of search, exclusions), number of studies focused on risk of incident dementia in people with late-life or midlife depression onset, dementia outcome, covariates controlled in individual analyses, pooled results (i.e., from meta-analyses) and key findings.

To explore the temporal relationship between depression and dementia, we extracted individual study data from included meta-analyses. To ensure completeness, we supplemented the electronic search of systematic reviews with hand-searching, including backward citation chasing and manual review of key study references, identifying additional primary studies missed by the reviews. Studies were included if they (1) clearly defined a timeframe for when depression was assessed i.e., Late-life depression was generally considered to occur in older adulthood (commonly defined as ≥65 years old), while midlife depression was considered to occur earlier in life (typically between 18 and 64 years old). However, we acknowledge that some studies adopted alternative thresholds for defining late-life depression, which were based on their specific study designs and interpretations (2) included a binary depression classification (i.e., depression present vs. absent); obtained through self-report, clinical diagnosis or determined using a cut-off score on a validated screener; (3) reported the risk of incident late-life all-cause dementia as a hazard ratio (HR); (4) were published from 2010 onwards, as to reflect current diagnostic criteria; and (5) studies were conducted in the general population. For each study, the first author, year of publication, total sample, number of incident cases, number of depression cases, follow-up time, model covariates, unadjusted and maximally adjusted individual study HR and corresponding 95% confidence intervals (CI) were extracted independently by two authors (JB, LG).

### Data analysis

Data were first synthesised using a narrative approach. A meta-meta-analysis was not undertaken due to overlapping primary publications included in multiple meta-analyses.

Meta-analyses of the results from the individual studies were conducted using Stata version 18.5. The natural logarithm of the HRs (logHR) and their corresponding confidence intervals (CI) were calculated to standardise the effect estimates. Results were back-transformed to the original scale (HRs) for ease of interpretation. Random-effects models were used to generate pooled estimates using the DerSimonian and Laird method.^16^ Where appropriate, subgroup analyses of adjusted models were conducted stratified by baseline age, follow-up duration, depression measure, geographical region, sample size, with specific adjustments for covariates such as the ε4 allele of the Apolipoprotein E genotype (APOE ε4), cardiovascular disease (CVD), diabetes, stroke, smoking status, and marital status. Subgroup analyses were performed only when the distribution of studies across the subgroups was balanced.

Heterogeneity was assessed using Cochran's Q-test and the *I*^*2*^ statistic.^17^ Publication bias was examined by visual inspection of funnel plots, Egger's regression,^18^ and the trim-and-fill method. To assess the robustness of results, sensitivity analyses were conducted using the leave-one-out method and a separate meta-analysis using unadjusted (crude) HRs.

Meta-regressions were conducted to explore sources of heterogeneity and impact of study-level covariates on observed associations. Covariates examined included mean age at baseline, mean follow-up time, total sample size, proportion of females, and number of dementia and depression cases. Studies variably used the terms “sex” and “gender” to describe participant characteristics, but none specified how these were determined (e.g., self-report or clinical records). For consistency, we used the reported proportion of female participants regardless of terminology. Three covariates were skewed (mean follow-up, proportion of females, number of depression cases) on visual inspections (e.g., histograms, Q–Q plots) and Shapiro–Wilk test for normality, necessitating log-transformation to normalise their distributions (results not published). Studies with missing data were excluded from the meta-regression analysis. Only univariable meta-regression models were employed when there was k ≥ 10 articles based on the ratio of 1 regression predictor per 10 studies. A bubble plot was generated to visualise the relationship between log follow-up time and dementia risk estimates.

### Role of the funding source

The funders of the study had no role in study design, data collection, data analysis, data interpretation, or writing of the report. All authors had access to the data used in this study, with authors JB, MA, and BCMS having the final responsibility for the decision to submit the study for publication.

## Results

**Fig. 1:**
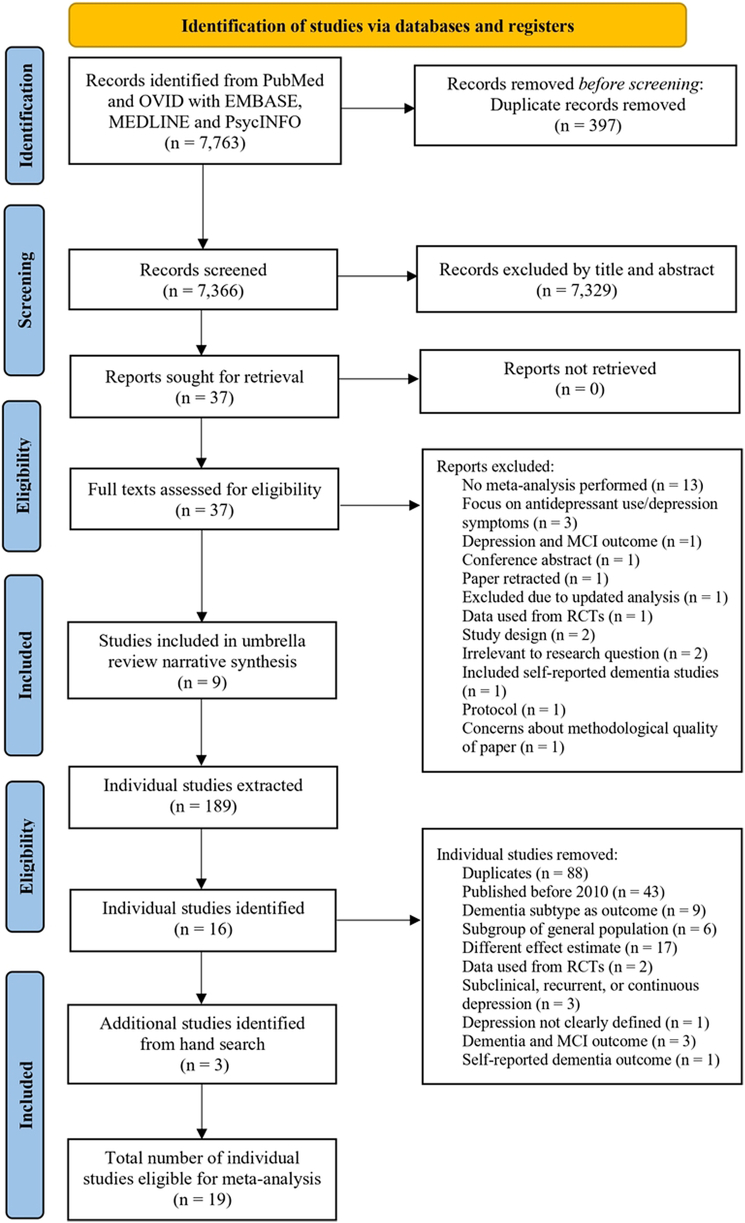
Flow diagram of study selection.

Of the 7763 articles screened, 37 were selected for full-text review. From these, eight fulfilled the inclusion criteria,^19^, ^20^, ^21^, ^22^, ^23^, ^24^, ^25^ with an additional article identified from backward citation chasing,^26^ resulting in nine reviews overall (Fig. 1).

### Umbrella review

Characteristics of the included reviews are shown in Table 1 (additional information provided in Appendix pp 3–5). Across reviews, the number of primary studies ranged from six to 36, with participants' baseline mean ages ranging from 52.7 to 83.8 years. Follow-up time ranged from one to 27 years. In most reviews, the endpoint was all-cause dementia (*n* = 8), followed by AD (*n* = 6), and VaD (*n* = 3). Three reviews investigated late-life depression and dementia risk,^19^^,^^20^^,^^24^ one review investigated depression across the lifespan and segregated early/midlife and late-life depression,^27^ while the remaining five reviews included primary studies focused on late-life and/or midlife depression but did not clearly state the timing of depression assessment in relation to dementia onset.^21^, ^22^, ^23^^,^^25^^,^^26^ There was large variability in depression classification, with some reviews including studies that used validated self-report measures, clinical diagnoses using operationalised criteria such as the DSM, or did not specify their inclusion criteria. No review was judged to be of high methodological quality. One review was rated as moderate quality^23^ with others rated critically low (*n* = 5)^19^^,^^21^^,^^25^^,^^26^ or low (*n* = 3)^20^^,^^22^^,^^24^ (Table 1; Appendix pp 6). Individual AMSTAR 2 domains of particular concern where 1 or fewer reviews were critically appraised as ‘yes’ related to explanations of study designs, comprehensive search strategies, citation to list of excluded studies with reasons, and sources of funding.

**Table 1:** Characteristics of included reviews**.**

Reference	Studies included in the final review	Search date (Databases)	Baseline age range of included studies (years)	Follow-up range of included studies (years)	Depression criteria	Timing of depression assessment	Dementia outcome(s)	AMSTAR-2
Cherbuin (2015)^19^	36	1996–2014 (PubMed, PsycINFO and Cochrane databases)	48–85	1–23.6	Depression diagnosed using a standardised assessment tool (excluded self-reported depression)	Defined as having depression at baseline, with title explicitly stating depression onset in late-life. No age parameters given.	All-cause, AD, VaD	Critically low
Diniz (2013)^20^	23	NR-2012 (PubMed and Scopus databases)	NR	1–17	Late-life depression (i.e., >50 years)	Late-life^a^	All-cause, AD, VaD	Low
Fernández (2023)^26^	26	2000–2022 (PubMed and PsycInfo)	Mean: 58.8–83.8	NR	NR	Midlife and late-life	All-cause	Critically low
Jorm (2001)^21^	13	Inception-2000 (Medline)	NR	1–21	NR	Midlife and late-life	All-cause	Critically low
Kuring (2020)^22^	31	1980–2019 (PubMed, EMBASE, PsycINFO and CINAHL)	65.5–88.8	2.4–20.2	Clinically significant levels of depression (clinical diagnosis using published diagnostic criteria)	Midlife and late-life	All-cause, AD, VaD	Low
Ownby (2006)^23^	20	NR (MEDLINE, PsychLit, EMBASE, and BIOSIS)	NR	NR	Diagnostic criteria that require the presence of symptoms consistent with major depressive disorder	Midlife and late-life	AD	Moderate
Saiz-Vazquez (2021)^24^	6	Inception-2020 (Web of Science, Scopus, PubMed, Elsevier Science Direct, and Google scholar)	52.7–81	1–23.6	Clinical diagnosis by established criteria (DSM-IV, ICD-10), or screening tool with a cut-off for clinically significant levels (e.g., Geriatric Mental State Schedule, GMS)	Late-life	AD	Low
Santabárbara (2020)^25^	8	2014–2019 (PubMed)	44.9–78.3	2–27	Validated instruments and scales, such as the CES-D (Centre of Epidemiological Studies Depression Scale)	Midlife and late-life	All-cause	Critically low
Stafford (2022)^27^	33	Inception-2021 (PubMed, PsycINFO, and Web of Science)	41.5–87.8	1–35	Depression had to meet diagnostic criteria through either clinical or validated self-report measures using validated thresholds	Midlife and late-life	All-cause	Low

Key: AD, Alzheimer's disease; AMSTAR-2, A Measurement Tool to Assess Systematic Reviews 2; CES-D, Center for Epidemiologic Studies Depression Scale; DSM-IV, Diagnostic and Statistical Manual of Mental Disorders, Fourth Edition; GMS, Geriatric Mental State Schedule; ICD-10, International Classification of Diseases, Tenth Revision; NR, Not reported; VaD, Vascular dementia.

a Authors criteria of late-life differed from the established criteria of this umbrella review.

Regarding late-life depression and risk of all-cause dementia pooled estimates ranged from RR 1.05 (95% CI: 1.02–1.08; all continuous measures of late-life depression) to RR 1.98 (95% CI: 1.50–2.63; all compatible categorically defined depression measures) (appendix pp7-9).^19^ Similarly, across the four reviews with an outcome of AD, pooled estimates ranged from RR 1.06 (95% CI: 1.02–1.10; all continuous measures of late-life depression)^19^ to OR 2.46 (95% CI: 1.81–3.35; depression diagnostic tool with a cut-off score).^24^ Two reviews investigated late-life depression and the risk of VaD,^19^^,^^20^ with pooled estimates ranging from RR 2.20 (95% CI: 0.8–5.59)^19^ to OR 2.52 (95% CI: 1.77–3.59).^20^ One review found no significant association with early/midlife depression and all-cause dementia midlife (RR 1.17, 95% CI: 0.80–1.73).

All reviews incorporating retrospective and prospective studies that did not specify the timing of depression onset found a significant, positive association with dementia,^21^, ^22^, ^23^^,^^25^, ^26^, ^27^ including (1) pooled estimates for all-cause dementia ranging from RR 1.63 (95% CI: 1.30–2.04)^25^ to RR 1.96 (95% CI: 1.59–2.43),^27^ and (2) pooled estimates ranged from OR 1.90 (95% CI: 1.55–2.33)^23^ to OR 2.23 (95% CI: 1.46–3.41)^22^ for AD. Meta-analyses of case–control studies reported that depression (irrespective of assessment timing) was associated with a significant increased risk of all-cause dementia (RR 2.01; 95% CI: 1.16–3.50)^21^ and AD (OR 2.03; 95% CI: 1.73–2.38).^23^

### Meta-analysis

For our meta-analyses, 19 individual studies were identified that assessed whether the timing of depression assessment impacted the strength of association with incident dementia.^28^, ^29^, ^30^, ^31^, ^32^, ^33^, ^34^, ^35^, ^36^, ^37^, ^38^, ^39^ All studies were considered good quality (Appendix pp 7).

**Fig. 2:**
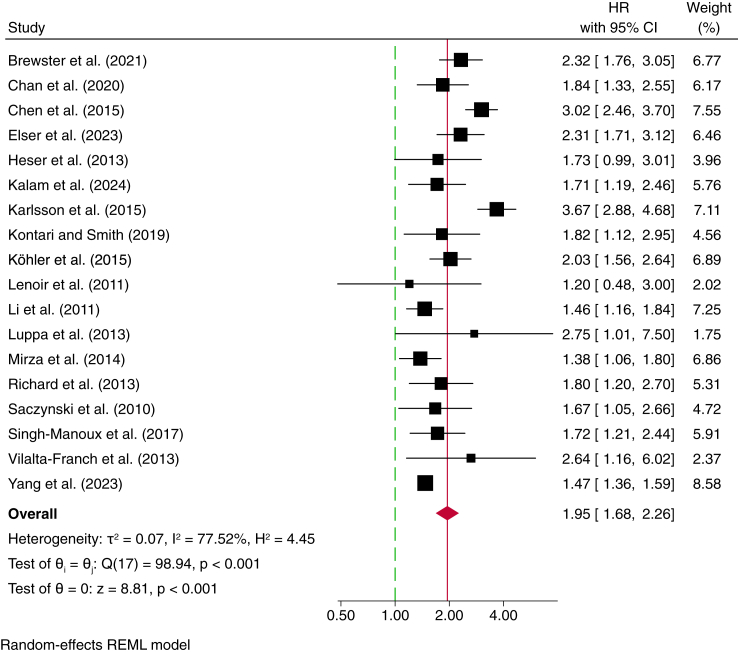
Random-effects meta-analysis of depression measured in late-life and the risk of all-cause dementia.

The meta-analysis evaluating the association between late-life depression and the risk of all-cause dementia included 18 studies,^28^, ^29^, ^30^, ^31^^,^^33^, ^34^, ^35^, ^36^, ^37^, ^38^, ^39^ with a total of 901,762 participants, 136,400 depression cases, and 7595 incident dementia cases (one study did not report depression cases^33^ and dementia cases^12^ (Table 2)). Data were derived from eleven European studies, four from North America, two from Asia, and one from Australasia. The overall pooled HR was 1.95 (95% CI, 1.68–2.26), with substantial heterogeneity (*I*^*2*^ = 77.5%) (Fig. 2). The funnel plot showed no clear asymmetry, and Egger's test indicated no statistically significant small-study effects (*p* = 0.9443), suggesting no strong evidence of publication bias (Appendix pp 8). The leave-one-out sensitivity analysis confirmed that no single study disproportionately influenced the pooled effect size, with effect estimates remaining stable across iterations (Appendix pp 9). A meta-analysis of crude (unadjusted) HRs also found a significant, positive association between late-life depression and the risk of all-cause dementia (HR 1.85; 95% CI: 1.42–2.42; *I*^*2*^ = 88.8%) (Appendix pp 10).

**Table 2:** Individual studies used **in** the meta-analysis investigating depression measured in late-life and all-cause dementia.

Author	Cohort (country)	Study design	Sample size (incident dementia cases)	N of females/women	Race/ethnicity	Mean age at baseline (SD)	Mean follow-up (years)	Depression measure	Age of depression assessment/diagnosis	N of depression cases	HR (95%CI)	Did authors consider depression at any other point of time (Yes/No)
Brewster et al. (2021)^28^	NACC (USA)	Cohort	8529 (498)	5412	Non-Hispanic White: 6583 Non-Hispanic African American: 1228 Hispanic: 493 Non-Hispanic Asian: 167 Other: 58	73.9 (7.9)	5.6	GDS	≥60 (just baseline)	613	2.32 (1.77–3.05)	No
Chan et al. (2020)^40^	NHIRD (Taiwan)	Cohort	16,725 (508)	5880^a^	NR	NR	NR	ICD-9-CM	≥65	3345	1.84 (1.33–2.55)	Yes
Chen et al. (2015)^29^	NHIRD (Taiwan)	Cohort	4582 (455)	1640	NR	65.34 (7.47)	4.01	ICD-9	≥55 (just baseline)	1946	3.02 (2.46–3.70)	No
Elser et al. (2023)^12^	DNPR (Denmark)	Cohort	445,038 (NR)	NR	NR	NR	NR	ICD	≥60	79,524	2.31 (2.25–3.12)	Yes
Heser et al. (2013)^30^	AgeCoDe (Germany)	Cohort	2663 (308)	1738	NR	81 (3.42)	4.5	DSM-IV	≥70 and they did not include those with a history of depression	57	1.73 (0.99–3.01)	Yes
Kalam et al. (2024)^41^	MAS (Australia)	Cohort	780 (195)	441	NR	78.1 (4.8)	NR (up to 12)	GDS	70–90	130	1.71 (1.19–2.46)	No
Karlsson et al. (2015)^11^	STR (Sweden)	Nested case–control	2404 (804)	NR	NR	79.5 (6.6)	NR	ICD codes, CES-D, and antidepressant prescription	≥60	388	3.67 (2.88–4.68)	Yes
Köhler et al. (2015)^42^	RNH (Netherlands)	Cohort	35,791 (1680)	18,951	NR	NR	NR (up to 13)	ICPC	≥50	978	2.03 (1.56–2.64)	Yes
Kontari and Smith, (2019)^31^	ELSA (UK)	Cohort	4859 (216)	2679^a^	NR	65.9 (9.37)	8	CES-D	≥50 (just baseline)	309	1.82 (1.13–2.95)	No
Lenoir et al. (2011)^33^	3C (France)	Cohort	7989 (276)	4900^a^	NR	74 (5.5)	4	MINI	≥60 (does not account for history)	NR	1.20 (0.50–3.00)	No
Li et al. (2011)^34^	ACT (USA)	Cohort	3410 (658)	2042^a^	NR	74.8 (6.2)	7.1	CES-D	≥50	342	1.46 (1.16–1.84)	No
Luppa et al. (2013)^35^	LEILA75+ (Germany)	Cohort	888 (183)	602^a^	NR	81.5 (5.3)	4.3	DSM-IV (SCID)	≥75	139	2.75 (1.01–7.50)	No
Mirza et al. (2014)^36^	RS (Netherlands)	Cohort	4393 (489)	2599^a^	NR	72.7 (7.3)	8.7	CES-D	Mean: 72.7	323	1.38 (1.06–1.80)	No
Richard et al. (2013)^43^	WHICAP (USA)	Cohort	1483 (207)	NR^a^	NR	76.9 (5.4)	5.4	CES-D	≥65	452	1.80 (1.20–2.70)	No
Saczynski et al. (2010)^37^	FHS (USA)	Cohort	736 (137)	NR	NR	79 (5.0)	17	CES-D or prescribed anti-depressant	Mean age 79	164	1.67 (1.05–2.66)	No
Singh-Manoux et al. (2017)^38^	Whitehall II (UK)	Cohort	6728 (177)	1980	NR for specific analysis	61.5 (6.0)	11.1	GHQ-30	50–74	1375	1.72 (1.21–2.44)	No
Vilalta-Franch et al. (2013)^39^	NR (Spain)	Cohort	451 (52)	295	NR	76.9 (5.5)	5	DSM-IV	≥65	35	2.64 (1.15–6.02)	No
Yang et al. (2023)^44^	UKBiobank (UK)	Cohort	354,313 (752)	189,440	White: 337,333 Non-white: 15,087	60.1 (5.4)	Median: 11.9	ICD-10	≥50	46,280	1.47 (1.35–1.59)	No

Key: ACT, Adult Changes in Thought Study; CES-D, Center for Epidemiologic Studies Depression Scale; CI, Confidence Interval; DNPR, Danish National Patient Registry; DSM-IV, Diagnostic and Statistical Manual of Mental Disorders, Fourth Edition; FHS, Framingham Heart Study; GDS, Geriatric Depression Scale; GHQ-30, General Health Questionnaire-30; HR, Hazard Ratio; ICD, International Classification of Diseases; ICD-9, International Classification of Diseases, Ninth Revision; ICD-10, International Classification of Diseases, Tenth Revision; LEILA75+, Leipzig Longitudinal Study of the Aged; MAS, Sydney Memory and Ageing Study; MINI, Mini International Neuropsychiatric Interview; NACC, National Alzheimer's Coordinating Center; NHIRD, National Health Insurance Research Database; NR, Not Reported; RNH, Registratie Net Huisartspraktijken; RS, Rotterdam Study; SCID, Structured Clinical Interview for DSM Disorders; STR, Swedish Twin Registry; UKBiobank, UK Biobank; WHICAP, Washington Heights-Inwood Columbia Aging Project; Whitehall II, Whitehall II Study; 3C, Three-City Study.

a Where studies used the term “gender” rather than “sex,” this is indicated in the table; however, none of the studies provided information on how sex or gender was determined (e.g., self-report, clinical records). One study (Mirza et al.) used these terms interchangeably.

Subgroup analyses (Appendix pp 11) showed variation in the association between late-life depression and dementia. Studies with ≤5.6 years follow-up had a stronger association (HR 2.21; 95% CI: 1.63–2.98) than those with >5.6 years (HR 1.69; 95% CI: 1.44–1.98), though the difference was not significant (*p* = 0.12). No significant differences were found when stratifying by mean age (<74.8 vs. ≥74.8 years, *p* = 0.62) or sample size (<3901.5 vs. ≥3901.5 participants, *p* = 0.79). Pooled estimates were similar between self-reported (HR 1.88; 95% CI: 1.52–2.34) and clinician-rated (HR 2.03; 95% CI: 1.64–2.50) depression measures (*p* = 0.64). Regionally, studies from North America (HR 1.79) and Europe (HR 1.95) showed comparable estimates, while the two studies from Asia had a notably larger effect (HR 2.40; 95% CI: 1.48–3.89), though the between-group difference was not significant (*p* = 0.68).

Subgroup analyses based on covariate adjustment showed minimal differences in pooled estimates (not shown). Studies not adjusting for APOE e4 had a slightly stronger association (HR 2.14; 95% CI: 1.68–2.72) than those that adjusted (HR 1.75; 95% CI: 1.51–2.03), though the difference was not significant (*p* = 0.16). Similarly, adjustment for CVD (HR 1.97 vs. 1.94, *p* = 0.94) and stroke (HR 1.95 vs. 1.95, *p* = 1.00) showed no meaningful differences. These analyses were balanced to match the subgroup comparisons, ensuring comparability across studies.

In the univariate meta-regression (Appendix pp 12), increasing follow-up duration was associated with a lower dementia risk estimate, explaining 44.58% of the variance (R^2^ = 44.58%) and reducing residual heterogeneity (*I*^*2*^ = 43.01%). Specifically, each unit increase in follow-up was associated with a decrease in HR (coefficient = −0.359, 95% CI: −0.695 to −0.022, *p* = 0.037). No other covariates were significantly associated with dementia risk estimates (*p* > 0.05). The bubble plot (Appendix pp 13) illustrates this association, with longer follow-up periods associated with lower dementia risk estimates, while shorter follow-up studies tended to estimate higher risks.

**Fig. 3:**
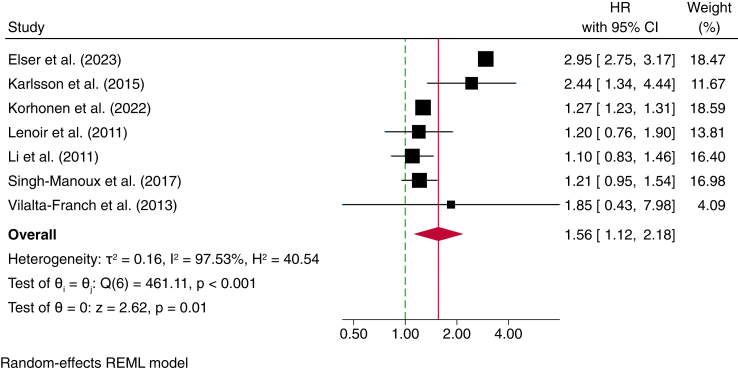
Meta-analysis of studies investigating depression measured in midlife and the risk of all-cause dementia.

Meta-analyses evaluating the association between depression measured in midlife and the risk of all-cause dementia included five studies (Table 3).^32^, ^33^, ^34^^,^^38^^,^^39^ A combined total of 2,501,296 participants were analysed, including 27,411 depression cases, and 276,929 incident dementia cases (some studies did not report number of depression or dementia cases in specific analyses^12^^,^^33^). The pooled HR suggested a 56% increased risk of all-cause dementia among individuals with midlife depression (HR 1.56; 95% CI: 1.12–2.18), with substantial heterogeneity (*I*^*2*^ = 97.5%) (Fig. 3). The funnel plot showed some asymmetry, though the small number of studies limits interpretability (Appendix pp 14). The leave-one-out sensitivity analysis (Appendix pp 15) indicated that removing individual studies had some impact on the pooled estimate, with the exclusion of one study^12^ resulting in the lowest pooled HR (1.27; 95% CI: 1.23–1.31) and reduced heterogeneity. However, the overall pattern remained consistent, supporting an association between midlife depression and increased dementia risk.

**Table 3:** Individual studies used in the meta-analysis investigating depression assessed in midlife and the risk of all-cause dementia.

Author	Cohort (country)	Study design	Sample size (incident dementia cases)	N of females	Ethnicity	Mean age at baseline (SD)	Age of depression assessment/diagnosis	Mean follow-up (years)	Depression measure	N of depression cases	HR (95% CI)	Did authors consider depression at any other point of time (Yes/No)
Elser et al. (2023)^12^	DNPR (Denmark)	Cohort	860,505 (NR)	NR	NR	NR	45–59	NR	ICD	NR	2.95 (2.75–3.17)	Yes
Karlsson et al. (2015)^11^	STR (Sweden)	Nested case–control	2404 (804)	939	NR	79.5 (6.6)	<60	NR	ICD codes, CES-D, and antidepressant prescription	36	2.44 (1.34–4.44)	Yes
Korhonen et al. (2022)^32^	Finish administrative healthcare data (Finland)	Cohort	1,616,321 (274,817)	903,027	NR	68.9 (6.2)^a^	∼39–54 (15–30 years prior to dementia follow-up)	9.1	ICD-9	23,959	1.27 (1.23–1.31)	No
Lenoir et al. (2011)^33^	3C (France)	Cohort	7989 (276)	4900	NR	74 (5.5)	<60	4	MINI	NR	1.20 (0.80–2.00)	No
Li et al. (2011)^34^	ACT (USA)	Cohort	3410 (658)	2042	NR	74.8 (6.2)	<50	7.1	CES-D plus single item question^b^	658	1.10 (0.83–1.47)	No
Singh-Manoux et al. (2017)^38^	Whitehall II (UK)	Cohort	10,189 (322)	3351	NR for specific analysis	45.1 (6.0)	35–55	26.6	GHQ-30	2744	1.21 (0.95–1.54)	No
Vilalta-Franch et al. (2013)^39^	NR (Spain)	Cohort	451 (52)	295	NR	76.9 (5.5)	<65	5	DSM-IV	14	1.85 (0.43–8.00)	No

Key: ACT, Adult Changes in Thought Study; CES-D, Center for Epidemiologic Studies Depression Scale; CI, Confidence interval; DNPR, Danish National Patient Registry; DSM-IV, Diagnostic and Statistical Manual of Mental Disorders, Fourth Edition; GHQ-30, General Health Questionnaire-30; HR, Hazard Ratio; ICD, International Classification of Diseases; MINI, Mini International Neuropsychiatric Interview; NR, Not Reported; STR, Swedish Twin Registry; 3C, Three-City Study.

a Mean age at follow-up period.

b Participants were asked the question “Have you ever had episodes of depression (feeling sad, blue, hopeless, or down in the dumps) lasting longer than two weeks?”

## Discussion

This umbrella review and meta-analysis systematically explored the timing of depression assessment and incident dementia, synthesizing findings from all previous systematic reviews and meta-analyses, which were generally of low quality. By combining and re-analysing the most up-to-date data, we provide a comprehensive assessment of how midlife and late-life depression contributes to dementia risk, offering novel insights into the temporality and magnitude of this association. The results highlighted a consistent relationship between late-life depression and risk for all cause dementia. Subgroup analyses demonstrated significant variability in effect sizes across different study characteristics including follow-up duration, sample size, and region. Studies with shorter follow-up durations and larger sample sizes generally reported stronger associations between late-life depression and dementia risk. The results suggest a strong association between depression and risk of dementia when depression occurs in late-life and is closer to the onset of cognitive decline, aligning with the 2020 Lancet Commission.^7^ However, the 2024 updated Commission re-classifies depression as a midlife risk factor, based on emerging evidence which is partially supported here.^33^

The relationship between late-life depression and dementia is complex and likely reflects multiple interacting factors. Depression in late life may represent an early manifestation of dementia, with common symptoms including impaired concentration, attention and other aspects of executive functioning, inertia, apathy, lassitude, and psychomotor agitation. Depression may also reflect underlying neurobiological changes that precede the clinical presentation of cognitive symptoms.^45^^,^^46^ Depression itself may accelerate cognitive decline through mechanisms linked to chronic inflammation, vascular dysregulation, and hypothalamic–pituitary–adrenal axis dysfunction, which further weaken neural resilience.^47^, ^48^, ^49^ In contrast, while there is evidence of a relationship between depression in earlier life stages and subsequent development of dementia, the limited number of studies and the heterogeneity regarding age stages and covariates make it challenging to draw definitive conclusions. For example, the observed heterogeneity in the late-life meta-analysis might reflect interactions with other factors such as vascular changes, neuroinflammation, disease co-morbidity.^50^ Additionally, the role of treatment, especially early detection and intervention for depression, may attenuate the risk of dementia onset and should be a focus of future research. Younger individuals may also have greater resilience and recovery potential due to higher neuroplasticity and cognitive reserve^51^ as well as increased opportunities for meaningful participation in occupational and social activities that support depression recovery. Survival bias could influence the observed relationship, as individuals with high dementia risk may not live long enough to develop the disease, as well as depression in younger life is less likely to be a prodromal symptom of dementia, unlike late-life depression, which may coincide with the early stages of cognitive decline.

A critical distinction in depression research is between self-reported symptoms and clinical diagnoses. Self-report measures may capture transient mood disturbances or symptoms unrelated to clinical depression, potentially overestimating depression prevalence.^52^ Clinical interviews provide context, distinguishing true depressive symptoms from general health issues or normal life stressors. This is particularly relevant in older adults, where depressive symptoms may overlap with early signs of cognitive decline.^53^ Furthermore, cohort study participants often differ from clinical populations seeking help for depression, potentially skewing results. Even identical scores on depression scales may represent different symptom profiles and underlying causes.^54^ These assessment differences likely contribute to the heterogeneity in reported associations between depression and dementia risk. Future research should prioritise standardised depression assessments, ideally combining self-report measures with clinical evaluations, to clarify whether clinical depression truly increases dementia risk or if certain depressive symptoms are prodromal to cognitive decline.

Several factors modified the association between depression and the risk of all-cause late-life dementia. For late-life depression, a shorter follow-up duration was linked to a stronger association with dementia risk, suggesting that depressive symptoms occurring closer to dementia onset may reflect prodromal features of dementia. Only two late-life study utilised a geriatric depression measure,^28^^,^^41^ which may more accurately represent the experience of late life depression including social withdrawal, apathy, and boredom without measuring overt somatic or physical symptoms.

These findings have significant implications for clinical practice and public health, highlighting the importance of managing depression across the life-course. Our results support the inclusion of depression in population attributable risk models for the global burden of dementia.^4^ Considering regional disparities in depression prevalence and dementia incidence in the Global Burden of Disease studies, targeted efforts are essential in areas with the highest needs. While the 2024 Commission shifted focus toward midlife depression, our findings suggest that late-life depression remains a critical period of dementia risk, and thus, identification and intervention for depression is required at both life stages. For individuals with depression, opportune access to effective pharmacological and non-pharmacological treatments, such as psychological therapies, may mitigate the risk of progression to dementia.^44^ Our findings suggest that although depression emerging near dementia onset may partly reflect prodromal changes, it nonetheless represents a potentially modifiable risk factor, as timely and effective intervention could mitigate the progression of cognitive decline. Future research should therefore prioritise controlled intervention studies to evaluate whether depression treatment reduces dementia risk, complemented by standardised longitudinal investigations incorporating neuroimaging and biomarker assessments to elucidate the underlying mechanisms of this association. Moreover, research is also needed to test whether interventions that promote physical activity and other lifestyle changes as strategies to support mental health treatment and recovery can support dementia risk reduction.^55^

Key study strengths include a comprehensive literature search and umbrella review that encompassed non-English publications. By extracting individual study data from previous meta-analyses, we standardised effect estimates, enhancing the robustness of our analysis. Detailed subgroup and meta-regression analyses allowed us to explore the influence of various covariates on the relationship between late-life depression and incident dementia, providing a nuanced understanding of how the timing of depression assessment affects dementia risk.

There are some limitations. First, the exclusion of studies due to incomplete data may affect the generalisability of our results. Second, there was significant variability in how studies defined midlife and late-life depression, with differing age thresholds and criteria across studies. Depression onset, severity, chronicity, recurrence, treatment and remission were poorly defined by all studies. This complicates direct comparisons and contributes to statistical and methodological heterogeneity. Third, some studies ascertained midlife depression retrospectively using a single self-reported question, introducing potential recall bias and concerns about the reliability and validity of the depression assessments. Additionally, in studies examining late-life depression, some did not account for previous episodes of depression throughout the life-course, potentially confounding the observed association due to unmeasured earlier depression. Studies were also excluded due to unclear reporting of the timing of depression assessment, and thresholds for defining timing varied greatly across studies. Moreover, none of the included studies reported how sex or gender were determined (e.g., self-report, clinical records), which may affect the accuracy and interpretation of sex-related findings in our analyses. The overall quality of included reviews was low, with eight of the nine reviews rated as low or critically low, which further limits confidence in some pooled estimates. While GRADE has been adapted for use in prognostic research, we determined it was not feasible in this umbrella review due to the high heterogeneity in study design, definition of depression, and variability in quality across included meta-analyses. Instead, we relied on AMSTAR 2 and NOS tools for appraisal and conducted a range of additional sensitivity and subgroup analyses to strengthen the robustness and interpretability of our findings. Most individual studies also lacked ethnic/racial data, constraining subgroup exploration. Finally, our analyses of midlife depression were limited to seven studies, precluding subgroup analyses, meta-regression, and reliable publication bias assessments.

This umbrella review and meta-analysis demonstrates that depression across the life course, particularly in later life, is associated with an increased risk of dementia, suggesting it may function both as a prodromal symptom and a modifiable risk factor. This highlights the potential need for targeted screening and depression management in older adults especially. However, further research is needed, especially focusing on earlier life stages, to understand how factors such as the moderating effect of treatment, patterns of recurrence or remission, and the life course characteristics of depression, including the number of episodes, chronicity, and severity affect dementia risk. Severity is likely to play an important role but was not captured in this analysis. Additionally, studies conducted in low- and middle-income countries, as well as those outside of North America and Europe, are essential for developing effective global prevention strategies and ensuring broader representation in dementia research.

## Contributors

JB, MA and BCMS designed the study. Article screening was done by JB, MA, MS, EYHT, and BCMS. Data extraction was undertaken by JB, MA, AHK, LG, and BCMS. Critical appraisal was done by JB, AHK, and LG. All data analyses were done by JB and MA. JB, MA, and BCMS have access to and verify the underlying study data. Supervision of the data analysis was done by MS, PJT, and BCMS. A first draft of the manuscript was produced by JB and MA. All authors contributed to the final version and approved all content.

## Data sharing statement

All data in this review were from publicly available systematic reviews and meta-analyses and reported in the original studies. All data synthesised as effect sizes in the current umbrella review and meta-analysis are reported in the table and figures. Please contact the corresponding author for further information regarding the data used in the analysis.

## Declaration of interests

PT received consulting fees from Pearson Australia for expert review, travel support from the Australian Cardiovascular Rehabilitation Association (ACRA) and the SOLD CHD Medical Research Future Fund, payment for advisory board participation from the National Heart Foundation of Australia, and payment for editorial board membership (Heart Mind, Wolters Kluwer) and grant assessment (Medical Research Future Fund). EB received a grant from the NHMRC Investigator Grant. BCMS's Chair in Dementia is co-funded by Curtin University and Dementia Australia. EYHT received support from an NIHR Clinical Lectureship. JB, LG, MS, AN, BM, AHK, MA, and DT declare no competing interests.
