# Supplementary material for: Temporal dynamics in the association between depression and dementia: an umbrella review and meta-analysis
Source: eClinicalMedicine. 2025 May 29;84:103266. doi: 10.1016/j.eclinm.2025.103266 (PMC12273843; doi:10.1016/j.eclinm.2025.103266)

**Appendix materials**

**Article Title**

Temporal Dynamics in the Association Between Depression and Dementia: An Umbrella Review and Meta-analysis

**Appendix 1** Search strategy

**Appendix 2** Table of meta-analysis results of the included reviews

**Appendix 3** AMSTAR 2

**Appendix** **4** Newcastle-Ottawa Scale critical appraisal

**Appendix** **5** Late-life depression and incident dementia funnel plot

**Appendix 6** Late-life depression and incident dementia leave-one-out sensitivity analysis

**Appendix 7** Late-life depression and incident dementia crude meta-analysis

**Appendix 8** Late-life depression and incident dementia subgroup analyses

**Appendix** **9** Late-life depression and incident dementia univariate meta-regression

**Appendix 10** Bubble plot visualising the relationship between log follow-up time and logHR in meta-regression

**Appendix 11** Midlife depression and incident dementia funnel plot

**Appendix 12** Midlife depression and incident dementia leave-one-out sensitivity analysis

**Appendix 1** Search strategy

| **Database** | **Search** | **Filters applied** | **Results** |
| --- | --- | --- | --- |
| PubMed from inception to 2025 Feb 17 | (depress* or mood) and (dementia OR alzheimer OR vascular) | Systematic review  Meta analysis | N=1,190 |
| OVID**  **MEDLINE** from 1946 to 2025 Feb 17  **Embase**1974 to 2025 Feb 17  **APA PsycArticles** 1967 to February Week 2 2025  **used basic search with related terms | (Depression OR depressed) AND (Dementia OR Alzheimer OR vascular) AND (Systematic Review OR meta){Including Limited Related Terms  Search terms included   - Depression - Depressed - Dementia - Alzheimers - Vascular - Systematic review - Meta analysis | Reviews | N=6,573 |
|  |  | TOTAL | 7,763 |
|  |  | TOTAL _excluding duplicates_ | 7,366 |


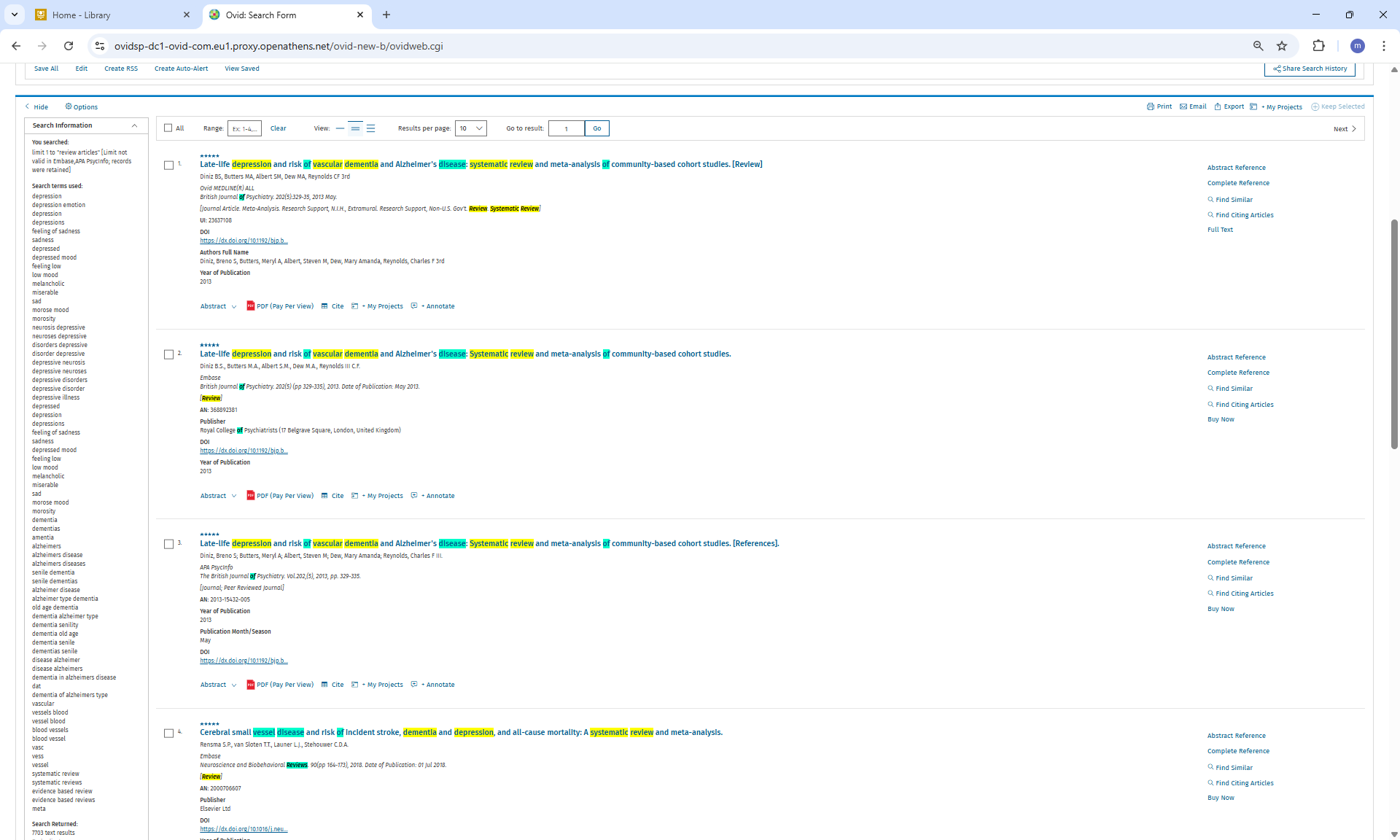

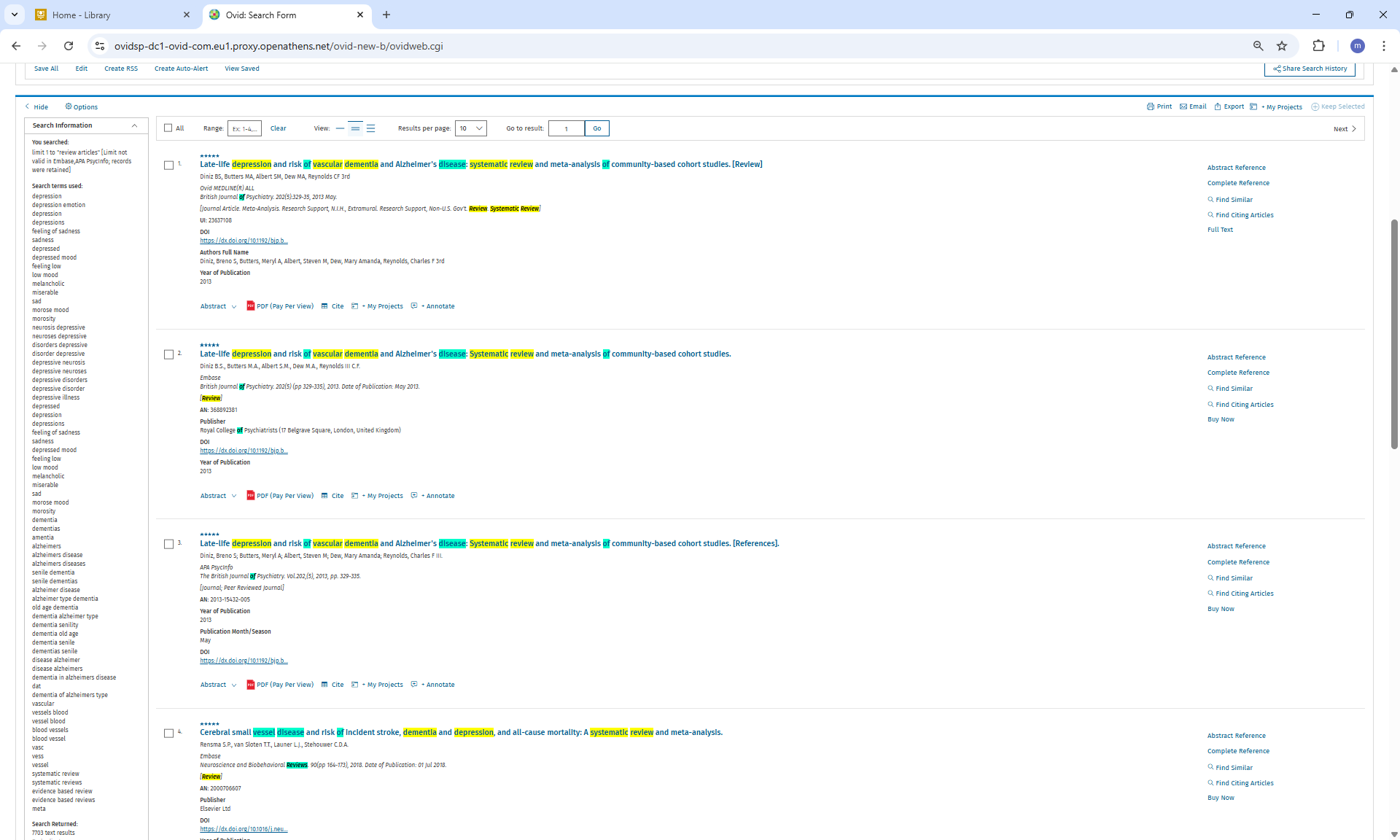


**Appendix 2** Table of meta-analysis results of the included reviews

| **Reference** | **Outcome** | **Included study design** | **Depression timing** | **Sample size** | **Total number of cases/events** | **Number of primary studies** | **Follow-up** | **Effect size metric** | **Meta-analysis method** | **Summary effect estimate** | **95% confidence interval** | **P value of summary effect estimate** | **Heterogeneity** | **Largest individual study** | **Suggestions of bias in relevant tests** |
| --- | --- | --- | --- | --- | --- | --- | --- | --- | --- | --- | --- | --- | --- | --- | --- |
| Cherbuin (2015) | AD (continuous – all compatible) | Prospective | Late-life | NR | NR | 10 | NR | RR | Random-effects | 1.06 | 1.02 – 1.10 | NR | I^2^ = 62.06% | 1.05 (1.01 – 1.09) | They suggest some publication bias is likely to be present and the actual effect-size smaller than reported. |
|  | AD (continuous – CES-D/HAM) | Prospective | Late-life | 4401 | NR | 6 | Mean: 5.9 | RR | Random-effects | 1.06 | 1.00 – 1.12 | NR | I^2^ = 72.87% | 1.06 (1.03 – 1.10) |  |
|  | AD (clinical threshold – all compatible) | Prospective | Late-life | NR | NR | 10 | NR | RR | Random-effects | 2.04 | 1.40 – 2.98 | NR | I^2^ = 60.28% | 1.24 (0.78 – 1.97) |  |
|  | AD (clinical threshold – CES-D >20) | Prospective | Late-life | 7831 | NR | 5 | Mean: 6.3 | RR | Random-effects | 1.97 | 0.96 – 4.04 | NR | I^2^ = 70.96% | 2.38 (1.15 – 4.93) |  |
|  | AD (clinical threshold – CES-D >16) | Prospective | Late-life | NR | NR | 6 | NR | RR | Random-effects | 1.58 | 1.25 – 2.00 | NR | I^2^ = 0.00% | 1.43 (1.05 – 1.94) |  |
|  | All-cause (continuous – all compatible) | Prospective | Late-life | NR | NR | 10 | NR | RR | Random-effects | 1.05 | 1.02 – 1.08 | NR | I^2^ = 63.06% | 1.03 (1.01 – 1.05) |  |
|  | All-cause (continuous – CES-D/HAM) | Prospective | Late-life | 8644 | NR | 8 | Mean: 5.2 | RR | Random-effects | 1.05 | 1.02 – 1.07 | NR | I^2^ = 54.82% | 1.03 (1.01 – 1.05) |  |
|  | All-cause (categorical – all compatible) | Prospective | Late-life | NR | NR | 11 | NR | RR | Random-effects | 1.98 | 1.50 – 2.63 | NR | I^2^ = 38.34% | 2.20 (1.30 – 3.71) |  |
|  | All-cause (categorical – CES-D >20) | Prospective | Late-life | 15,553 | NR | 5 | Mean: 8.2 | RR | Random-effects | 1.83 | 0.95 – 3.52 | NR | I^2^ = 71.85% | 0.70 (0.39 – 1.26) |  |
|  | All-cause (categorical – CES-D >16) | Prospective | Late-life | NR | NR | 9 | NR | RR | Random-effects | 1.69 | 1.46 – 1.97 | NR | I^2^ = 0.00% | 1.71 (1.37 – 2.13) |  |
|  | VaD (continuous – all) | Prospective | Late-life | 3187 | 37 | 3 | NR | RR | Random-effects | 1.06 | 0.92– 1.21 | NR | NR | 1.11 (1.03 – 1.18) |  |
|  | VaD (categorical – all) | Prospective | Late-life | 13,331 | 113+ | 2 | NR | RR | Random-effects | 2.20 | 0.87 – 5.59 | NR | NR | 1.78 (0.98 – 3.23) |  |
| Diniz (2013) | All-cause | Prospective | Late-life | 49,612 (5,116 with late-life depression, 44,496 controls) | NR | 25 | Median: 5 | OR | Random-effects | 1.85 | 1.67 – 2.04 | <0.001 | I^2^ = 0.00% | 1.72 (1.38 – 2.13) | Visual inspection of funnel plots indicated no publication bias |
|  | AD | Prospective | Late-life | 28,746 (3,437 with late-life depression, 25,309 controls) | NR | 16 | Median: 5 | OR | Random-effects | 1.65 | 1.42 – 1.92 | <0.001 | I^2^ = 2.00% | 1.43 (1.05 – 1.96) |  |
|  | VaD | Prospective | Late-life | 14,901 (1,801 with late-life depression, 13,100 controls) | NR | 5 | Median: 6.1 | OR | Random-effects | 2.52 | 1.77 – 3.59 | <0.001 | I^2^ = 2.00% | 1.79 (0.99 – 3.22) |  |
| Fernández (2023) | All-cause | Prospective | Any | 1,760,262 | NR | 26 | Range: 3-17 | HR | Random-effects | 1.82 | 1.62–2.06 | <0.005 | I^2^ = 95.33% | 3.68 (2.10 - 6.43) | Egger’s test not significant |
| Jorm (2001) | All-cause | Case-control | Any | NR | NR | 7 | NR | RR | NR | 2.01 | 1.16 – 3.50 | NR | NR | 1.80 (0.90 – 3.50) | NR |
|  | All-cause | Prospective | Any | NR | NR | 7 | Range: 1-10 | RR | NR | 1.87 | 1.09 – 3.20 | NR | NR | 2.38 (1.08 – 5.06) |  |
| Kuring (2020) | All-cause | Prospective and retrospective | Any | 446,283 | NR | 31 | Range:2.4-20.2 | OR | Random-effects | 1.91 | 1.72 – 2.12 | <0.001 | I^2^ = 64.69% | 1.85 (1.77 – 1.92) | Trim-and-fill procedure revealed current estimate of all-cause dementia development (associated with prior depression) was likely to be missing from 10 studies 1.73(95% CI 1.54–1.95). |
|  | AD | Prospective and retrospective | Any | 16,691 | NR | 8 | Range:3.5-6.1 | OR | Random-effects | 2.23 | 1.46 – 3.41 | <0.001 | I^2^ = 72.49% | 1.22 (0.89 – 1.67) |  |
| Ownby (2006) | AD | Case-control | Any | 7,068 | NR | 9 | NR | OR | Random-effects | 2.03 | 1.73 – 2.38 | <0.001 | NR | 2.08 (1.69 – 2.56) | NR |
|  | AD | Prospective and retrospective | Any | 95,104 | NR | 11 | NR | OR | Random-effects | 1.90 | 1.55 – 2.33 | <0.001 | NR | 2.25 (2.00 – 2.54) | NR |
|  | AD | Case-control/cohort combined | Any | 102,172 | NR | 20 | NR | OR | Random-effects | 2.02 | 1.80 – 2.26 | <0.001 | NR | 2.25 (2.00 – 2.54) | Egger’s test, t=2.75; P=.01 |
| Santabárbara (2020) | All-cause | Prospective | Any | 2,484,976 | 61,759 | 8 | Range: 2-27 | RR | Random-effects | 1.63 | 1.30 – 2.04 | <0.001 | I^2^ = 95.3% | 1.68 (1.65 – 1.72) | Visual inspection of funnel plot may suggest presence of publication bias. However, Egger (p= 0.723) and Begg test p= 0.902) indicate minimal/no risk of bias. |
| Saiz-Vazquez (2021) | AD | Longitudinal (not specified) | Late-life | 101,881 | NR | 28 | Mean: 4.9  Range: 1-23.6 | OR | Random-effects | 2.46 | 1.81 – 3.35 | <0.001 | I^2^ = 90.51% | 4.15 (3.49 – 4.94) | Egger’s test not significant: intercept (B0) was 0.53, 95% CI (−1.88 to 2.95), with t = 0.45, df = 26, p = 0.65, indicating no publication bias |
| Stafford (2022) | All-cause | Longitudinal cohort (including case-cohort and nested case-control) | Any | NR | NR | 27 | Range 1-35 | RR | Random-effects | 1.96 | 1.59 – 2.43 | <0.001 | I^2^ = 96.5% | 1.11 (0.87-1.16) and 2.47 (2.35-2.58) | Visual inspection of funnel plot indicated no small study effects; Egger’s test, p = 0.45 |
|  | All-cause | Longitudinal cohort (including case-cohort and nested case-control) | Midlife | NR | NR | 6 | NR | RR | Random-effects | 1.17 | 0.80 – 1.73 | 0.051 | I^2^ = 54.6% | 1.10 (0.83-1.47) | NR |
|  | All-cause | Longitudinal cohort (including case-cohort and nested case-control) | Late-life | NR | NR | 5 | NR | RR | Random-effects | 1.92 | 1.13 – 2.34 | <0.001 | I^2^ =87.8% | 1.46 (1.16-1.84) | NR |
|  | AD | Longitudinal cohort (including case-cohort and nested case-control) | Any | NR | NR | 13 | NR | RR | Random-effects | 1.90 | 1.52 – 2.38 | <0.001 | I^2^ = 85.5% | 1.79 (1.68-1.92) | Egger’s test, p = 0.6 |
|  | VaD | Longitudinal cohort (including case-cohort and nested case-control) | Any | NR | NR | 6 | NR | RR | Random-effects | 2.,71 | 2.48 – 2.97 | 0.535 | I^2^ = 0.00% | 2.68 (2.44-2.95) | Egger’s test, p = 0.5 |

AD=Alzheimer's Disease; CES-D=Center for Epidemiologic Studies Depression Scale; CI=Confidence Interval; HAM=Hamilton Depression Rating Scale; HR=Hazard Ratio; NR=Not Reported; OR=Odds Ratio; RR=Relative Risk; VaD=Vascular Dementia

**Appendix 3** AMSTAR 2 critical appraisal


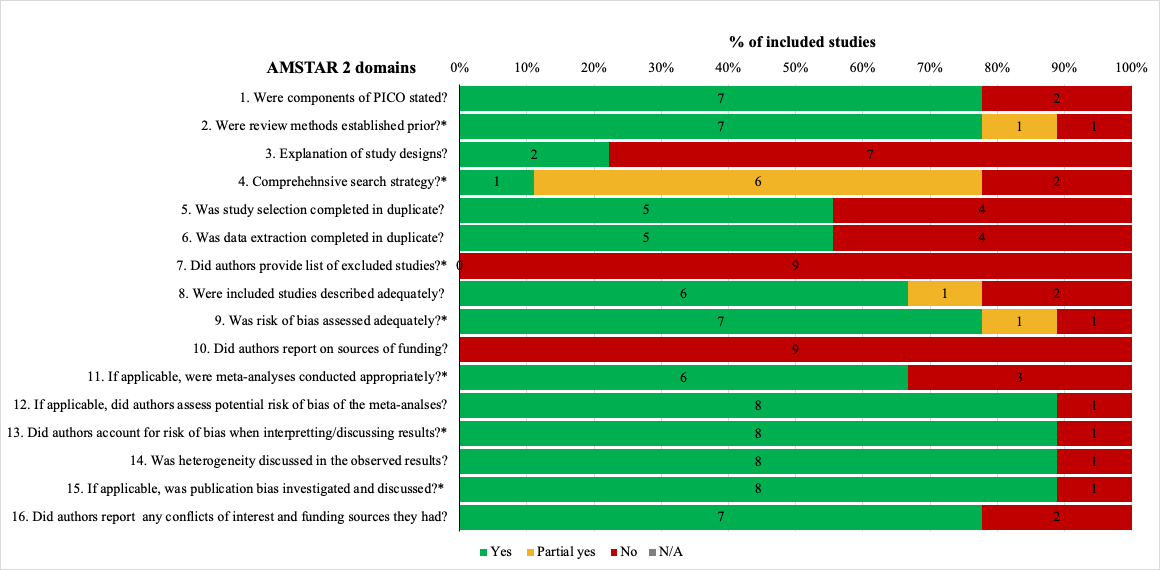


**Appendix** **4** Newcastle-Ottawa Scale critical appraisal of cohort studies

| **Author** | **Selection** | **Comparability** | **Outcome** | **Overall Risk of Bias Assessment** |
| --- | --- | --- | --- | --- |
| Brewster et al. (2021) | ★★★★ | ★★ | ★★★ | Good quality |
| Chen et al. (2015) | ★★★★ | ★★ | ★★★ | Good quality |
| Heser et al. (2013) | ★★★★ | ★★ | ★★★ | Good quality |
| Kontari and Smith (2019) | ★★★★ | ★★ | ★★★ | Good quality |
| Korhonen et al. (2022) | ★★★★ | ★★ | ★★★ | Good quality |
| Lenoir et al. (2011) | ★★★★ | ★★ | ★★★ | Good quality |
| Li et al. (2011) | ★★★★ | ★★ | ★★★ | Good quality |
| Luppa et al. (2013) | ★★★★ | ★★ | ★★★ | Good quality |
| Mirza et al. (2014) | ★★★★ | ★★ | ★★★ | Good quality |
| Saczynski et al. (2010) | ★★★★ | ★★ | ★★ | Good quality |
| Singh-Manoux et al. (2017) | ★★★★ | ★★ | ★★★ | Good quality |
| Vilalta-Franch et al. (2013) | ★★★★ | ★★ | ★★★ | Good quality |
| Yang et al. (2023) | ★★★★ | ★★ | ★★★ | Good quality |
| Elser et al. (2023) | ★★★★ | ★★ | ★★★ | Good quality |
| Köhler et al. (2015) | ★★★★ | ★★ | ★★★ | Good quality |
| Kalam et al. (2024) | ★★★★ | ★★ | ★★★ | Good quality |
| Richard et al. (2013) | ★★★ | ★★ | ★★★ | Good quality |
| Chan et al. (2020) | ★★★ | ★★ | ★★★ | Good quality |
| Karlsson et al. (2015) | ★★★★ | ★★ | ★★★ | Good quality |

Key

Good quality = 3/4 stars in ‘Selection’ domain, AND 1/2 stars in ‘Comparability’ domain, AND 2/3 stars in ‘Outcome’ domain.

 Fair quality = 2 stars in ‘Selection’ domain, AND 1/2 stars in ‘Comparability’ domain, AND 2/3 stars in ‘Outcome’ domain.

 Poor quality = 0/1 star in ‘Selection’ domain, OR 0 stars in ‘Comparability’ domain, OR 0/1 stars in ‘Outcome’ domain.

**Appendix** **5** Late-life depression and incident dementia funnel plot


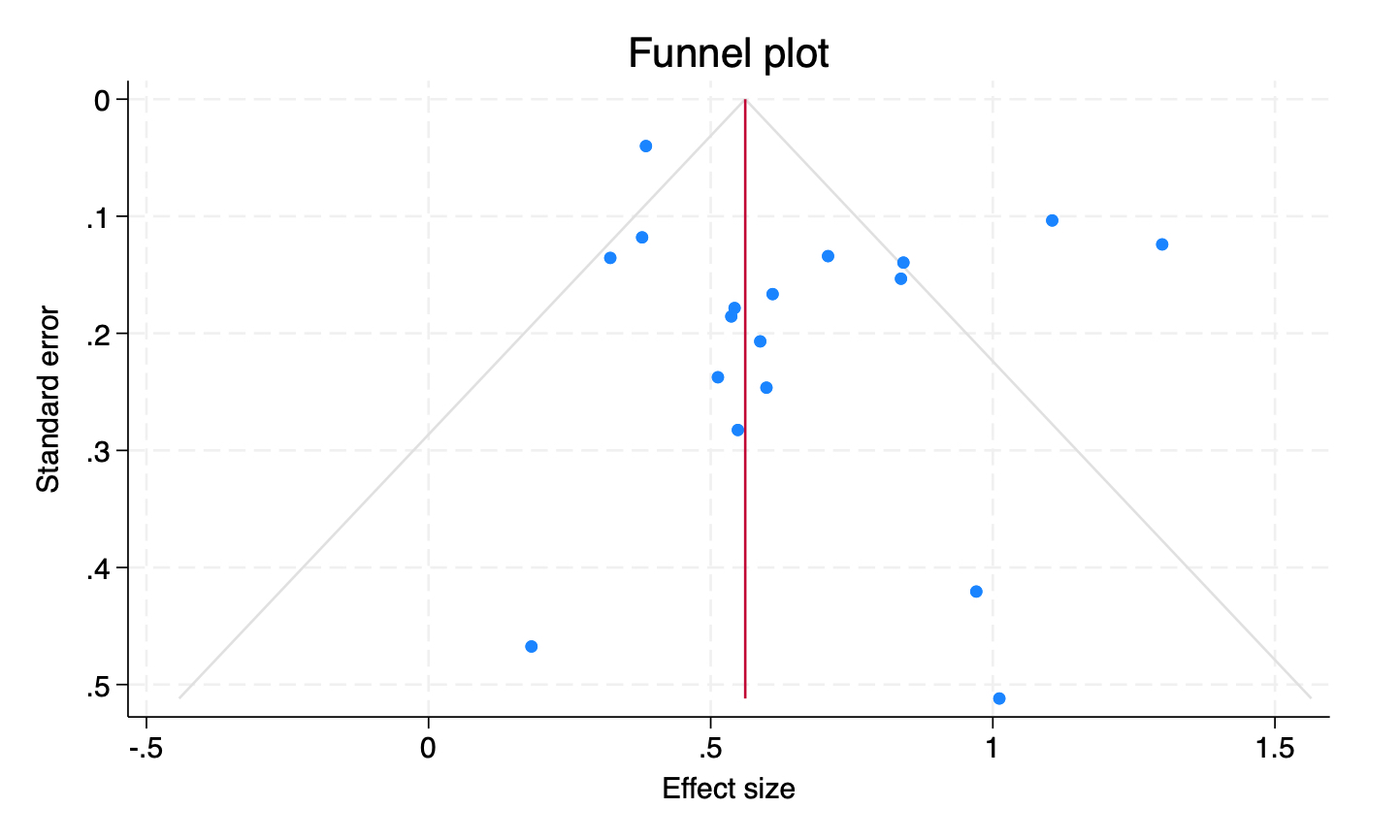


**Appendix 6** Late-life depression and incident dementia leave-one-out sensitivity analysis


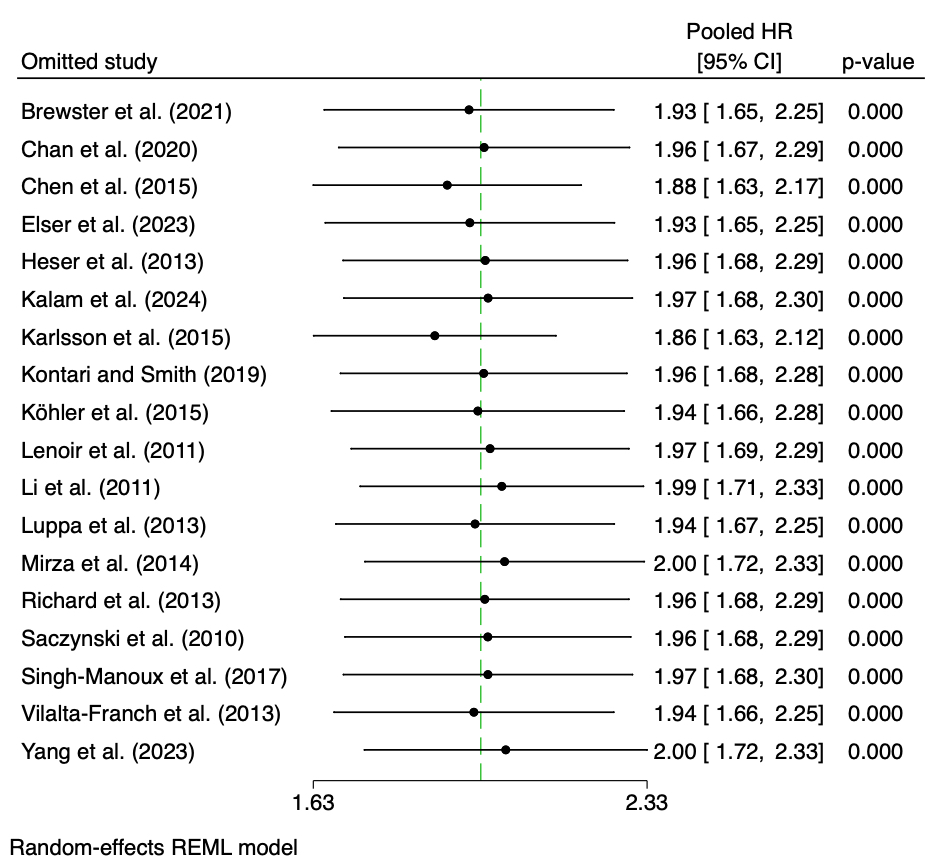


**Appendix 7** Late-life depression and incident dementia crude meta-analysis

**
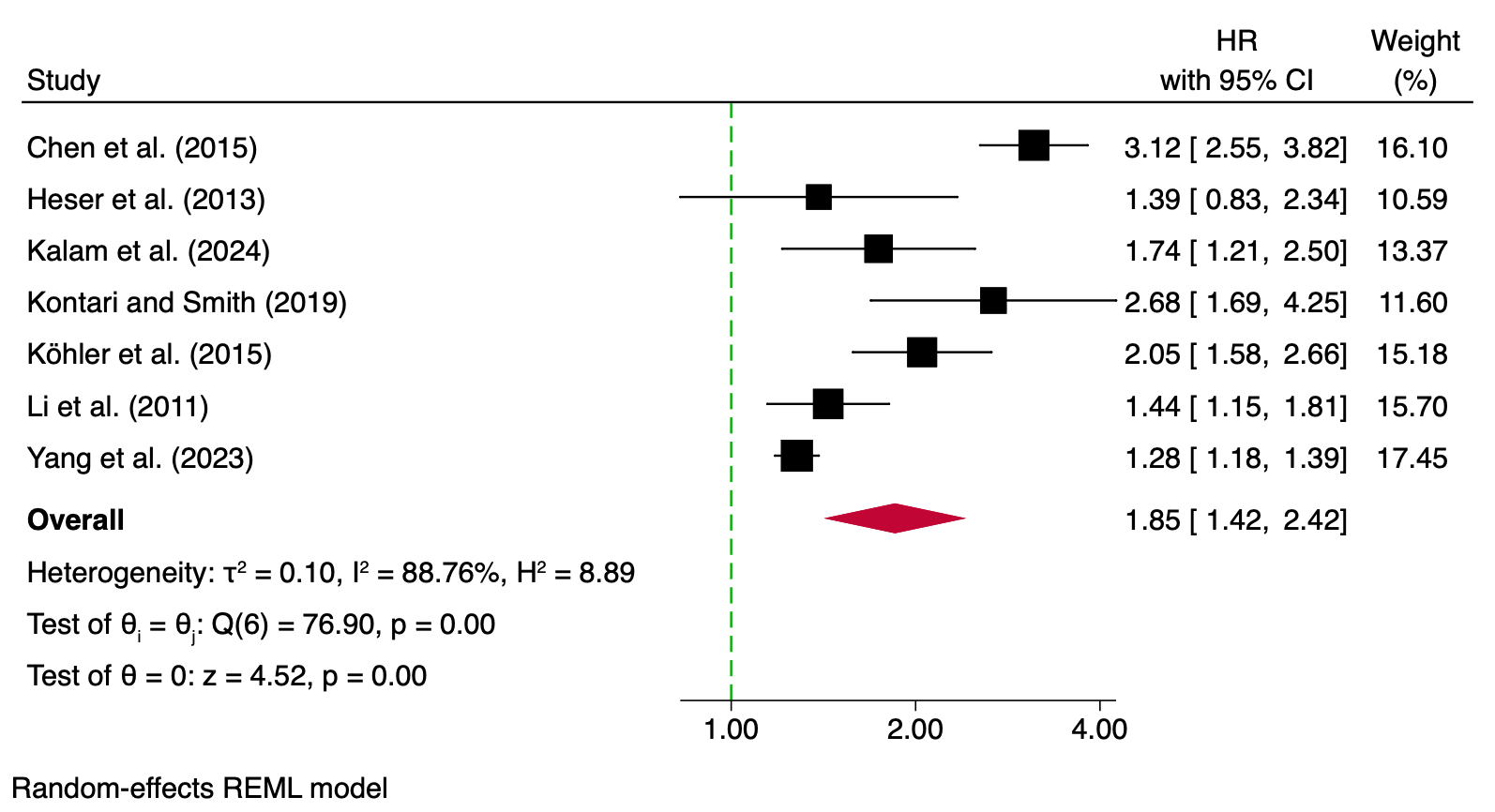
**

**Appendix 8** Late-life depression and incident dementia subgroup analyses

| **Subgroup** | **Number of studies (k)** | **Pooled HR (95% CI)** | **I^2^ (%)** | **p-value for between subgroup differences** |
| --- | --- | --- | --- | --- |
| **Study characteristics** | | | | |
| **Follow-up duration*** |  |  |  |  |
| < 5.6 years | 6 | 2.21 (1.63-2.98) | 47.8 | 0.12 |
| ≥ 5.6 years | 5 | 1.69 (1.44-1.98) | 39.2 |  |
| **Age group*** |  |  |  |  |
| < 74.8 years | 7 | 1.84 (1.43-2.36) | 84.8 | 0.62 |
| ≥74.8 years | 8 | 2.02 (1.54-2.65) | 70.8 |  |
| **Region** |  |  |  |  |
| North America | 4 | 1.79 (1.41-2.26) | 52.5 | 0.68 |
| Asia | 2 | 2.40 (1.48-3.89) | 84.3 |  |
| Europe | 11 | 1.95 (1.57-2.41) | 79.7 |  |
| Australasia | 1 | 1.71 (1.19-2.46) | - |  |
| **Depression measure** |  |  |  |  |
| Self-reported | 9 | 1.88 (1.52-2.34) | 41.7 | 0.64 |
| Clinician-rated | 9 | 2.03 (1.64-2.50) | 74.9 |  |
| **Sample size*** |  |  |  |  |
| < 3901.5 | 8 | 2.02 (1.54-2.65) | 70.8 | 0.79 |
| ≥ 3901.5 | 9 | 1.92 (1.58-2.33) | 81.0 |  |

*Subgroups categorised using the median values of included studies

Key

APOE=Apolipoprotein E; CI=Confidence Interval; HR=Hazard Ratio

**Appendix** **9** Late-life depression and incident dementia univariate meta-regression

| **Covariate** | **Number of included studies** | **Coefficient [95% CI]** | **Standard Error** | **p-value** | **R² (%)** | **I² (%)** | **Interpretation** |
| --- | --- | --- | --- | --- | --- | --- | --- |
| **Log mean follow-up** | 13 | -0.359 [-0.695, -0.022] | 0.172 | 0.037 | 44.58 | 43.01 | Longer follow-up → Lower HR |
| **Mean age at baseline** | 15 | 0.009 [-0.017, 0.035] | 0.013 | 0.500 | 0.00 | 76.52 | No association |
| **Log Female proportion** | 13 | -0.538 [-1.275, 0.200] | 0.376 | 0.153 | 17.31 | 80.73 | No association |
| **Log total sample size** | 16 | -0.020 [-0.108, 0.068] | 0.044 | 0.646 | 0.00 | 75.16 | No association |
| **Log depression cases** | 16 | -0.019 [-0.082, 0.043] | 0.032 | 0.548 | 0.00 | 76.95 | No association |
| **Dementia cases** | 16 | 0.0001 [-0.0003, 0.0005] | 0.0002 | 0.637 | 0.00 | 80.72 | No association |

*Univariate meta-regression analyses assessing the impact of study-level covariates on the association between late-life depression and dementia risk. Coefficients are presented with their 95% confidence intervals. R² represents the percentage of variance explained by each covariate, I² indicates the residual heterogeneity.*

**Appendix 10** Bubble plot visualising the relationship between log follow-up time and logHR in meta-regression

**
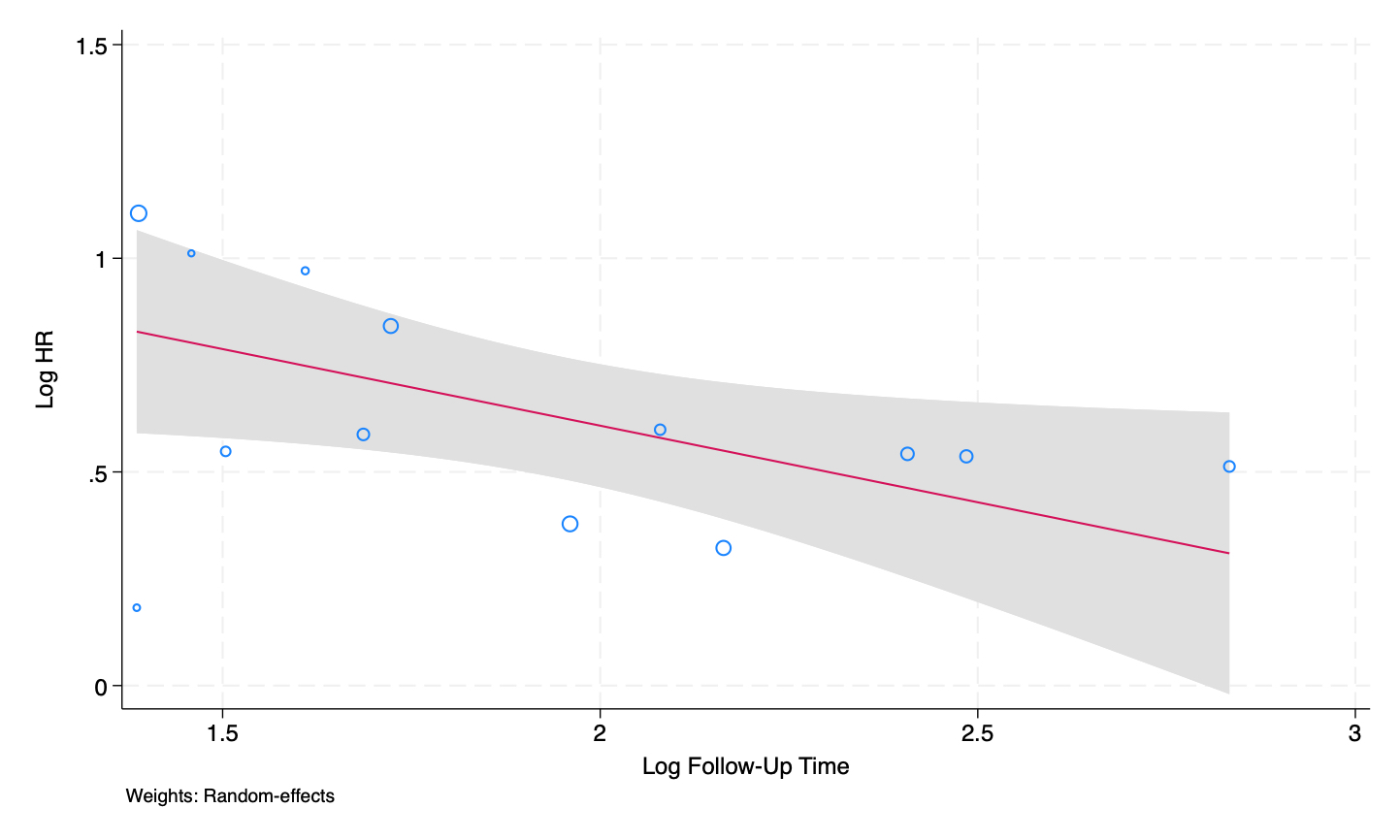
**

**Appendix 11** Midlife depression and incident dementia funnel plot


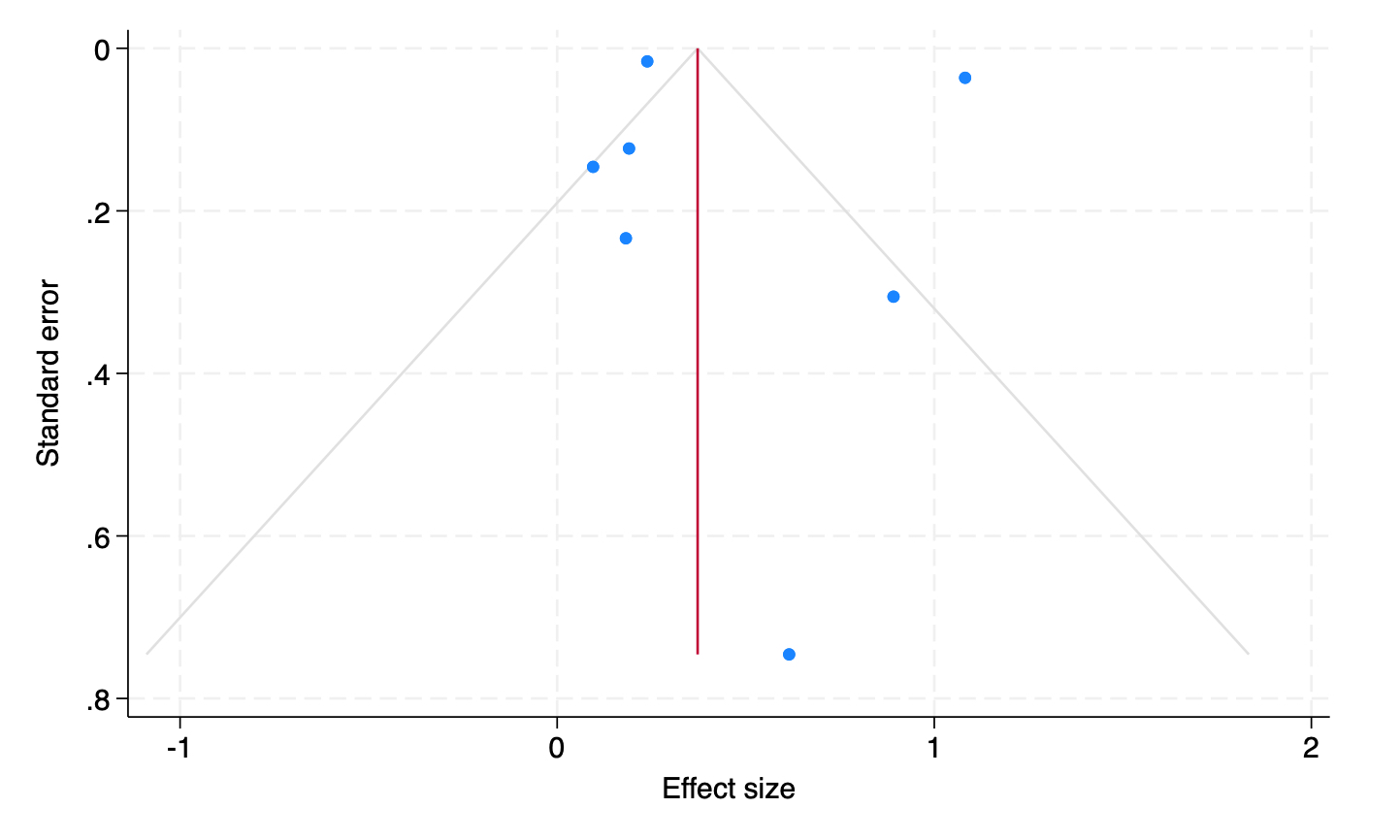


**Appendix 12** Midlife depression and incident dementia leave-one-out sensitivity analysis


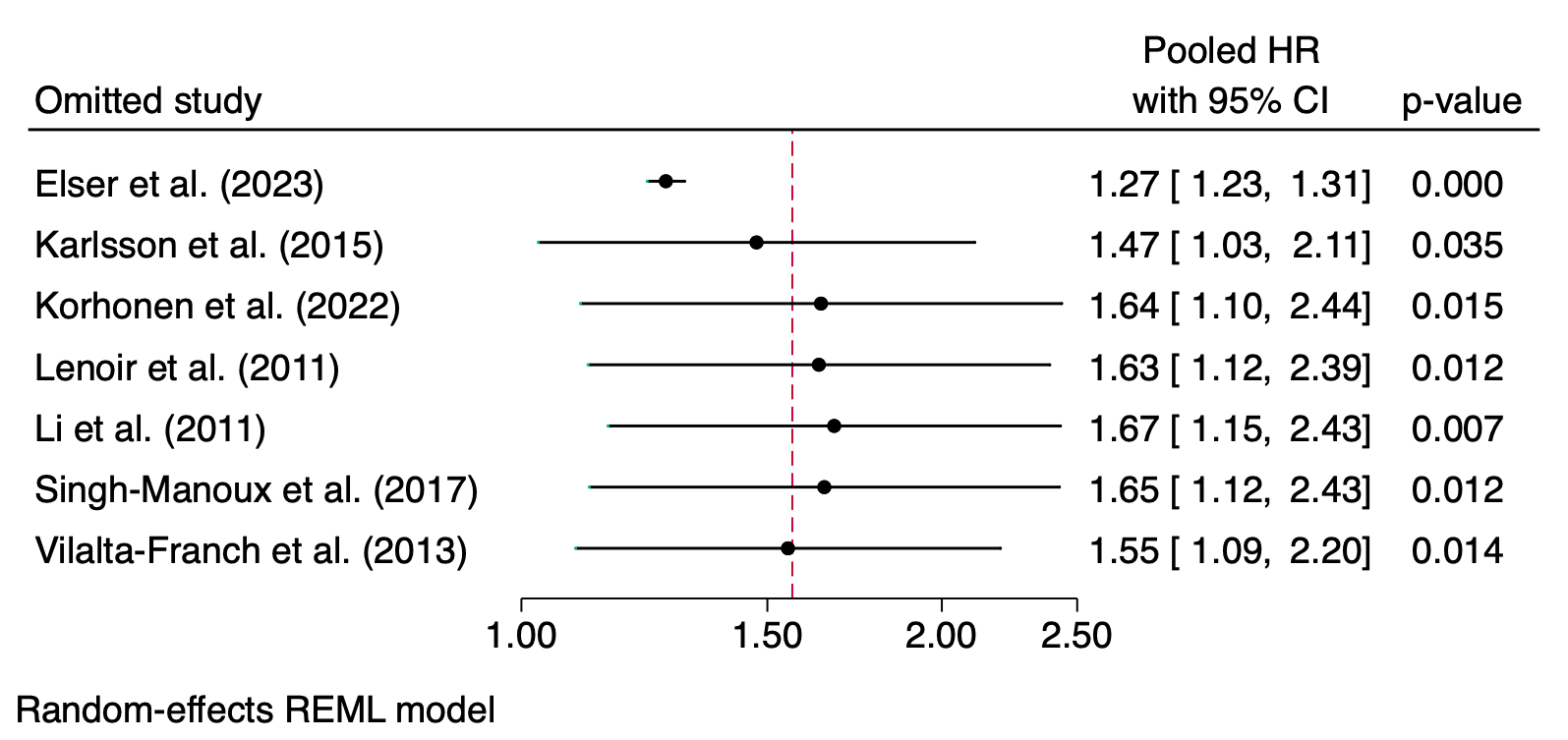

Supplement: Appendix [file mmc1.docx]
